# Uvaol Improves the Functioning of Fibroblasts and Endothelial Cells and Accelerates the Healing of Cutaneous Wounds in Mice

**DOI:** 10.3390/molecules25214982

**Published:** 2020-10-28

**Authors:** Julianderson Carmo, Polliane Cavalcante-Araújo, Juliane Silva, Jamylle Ferro, Ana Carolina Correia, Vincent Lagente, Emiliano Barreto

**Affiliations:** 1 Laboratory of Cell Biology, Federal University of Alagoas, 57072-900 Maceió, Brazil; julianderson.oliveira.bio@hotmail.com (J.C.); pollianearaujo@hotmail.com (P.C.-A.); juliane.silva@icbs.ufal.br (J.S.); jamylle.ferro@icbs.ufal.br (J.F.); 2 Garanhuns College of Science, Education and Technology, University of Pernambuco, 55294-902 Garanhuns, Brazil; ana.correia@upe.br; 3 NuMeCan Institute (Nutrition, Metabolism and Cancer), Université de Rennes, INSERM, INRA, F-35000 Rennes, France; vincent.lagente@univ-rennes1.fr

**Keywords:** uvaol, terpenes medicinal, wound healing, fibroblasts, endothelial cells

## Abstract

Uvaol is a natural pentacyclic triterpene that is widely found in olives and virgin olive oil, exerting various pharmacological properties. However, information remains limited about how it affects fibroblasts and endothelial cells in events associated with wound healing. Here, we report the effect of uvaol in the in vitro and in vivo healing process. We show the positive effects of uvaol on migration of fibroblasts and endothelial cells in the scratch assay. Protein synthesis of fibronectin and laminin (but not collagen type I) was improved in uvaol-treated fibroblasts. In comparison, tube formation by endothelial cells was enhanced after uvaol treatment. Mechanistically, the effects of uvaol on cell migration involved the PKA and p38-MAPK signaling pathway in endothelial cells but not in fibroblasts. Thus, the uvaol-induced migratory response was dependent on the PKA pathway. Finally, topical treatment with uvaol caused wounds to close faster than in the control treatment using experimental cutaneous wounds model in mice. In conclusion, uvaol positively affects the behavior of fibroblasts and endothelial cells, potentially promoting cutaneous healing.

## 1. Introduction

Wound healing is a dynamic and complex biological process that involves different types of cells working in concert to restore damaged tissues [1]. In this process, coordinated biological events, including phagocytosis, migration, proliferation, angiogenesis, and the synthesis of the extracellular matrix, culminates in the repair of injured tissue [2]. Indeed, wound healing begins at the moment of injury and progresses over three basic, highly integrated, and overlapping phases: (1) inflammation, which is associated with the recruitment of neutrophils and macrophages to the wound bed to clean the lesioned tissue; (2) tissue proliferation and formation, which involves the migration and proliferation of fibroblasts and the development of new blood vessels (angiogenesis); and (3) tissue remodeling, which involves the remodeling of the extracellular matrix to an architecture that approaches normal tissue [3,4].

Among biological processes involved in wound closure, the ability for cells to migrate to the wound bed is important. During the inflammatory phase, macrophages facilitate the non-phlogistic removal of various cell debris and secrete chemical mediators that promote the recruitment and activation of other cell types, including fibroblasts [5]. In turn, fibroblasts are responsible for producing the extracellular matrix, which ultimately forms the granulation tissue [6]. This matrix functions as a tissue support system, facilitating the migration of more fibroblasts and other cells, such as endothelial cells [7]. Endothelial cells require extracellular matrix (ECM) for migration to promote the formation of new blood vessels from the existing vasculature [2]. The formation of new blood vessels is a critical component of wound healing, with any impairment in the angiogenic response negatively impacting wound repair [8]. Therefore, fibroblasts and endothelial cells must function properly during the proliferation phase to prepare the foundation for the remodeling phase, which ensures tissue healing. Thus, improving the activity of these cells might help promote proper tissue repair and improve impaired healing. Therefore, it is important to identify compounds able to regulate the biological events of cells involved in healing, to develop new drugs aimed towards accelerated wound healing.

Plant-derived products have been used through the ages to treat various pathological processes, including wound healing [9,10]. These compounds induce healing and tissue regeneration through multiple connected mechanisms that often have synergistic effects on the overall efficiency of healing [11]. Amongst the many active components isolated from plants that have healing effects, pentacyclic triterpenes exhibit wound healing properties, mainly because they affect the production and activity of inflammatory mediators and growth factors [12,13].

**Figure 1:**
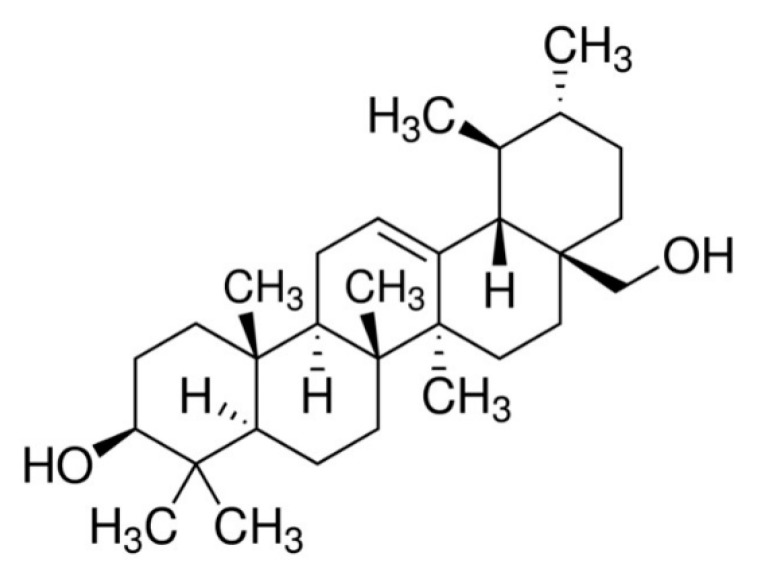
Chemical structure of uvaol.

Uvaol is a pentacyclic triterpene (Figure 1) that is abundant in olives and the leaves of olive trees (*Olea europaea*) [14]. It possesses a wide spectrum of pharmacological effects, including antioxidant [15] and anti-inflammatory activity [12]. In particular, uvaol interferes with allergic inflammatory responses by affecting the recruitment of leukocytes and production of cytokines at the site of inflammation [16]. It also has a vasodilator effect [17], inducing changes to the expression of surface proteins associated with cell adhesion [18]. The broad spectrum of pharmacological properties associated with this compound has resulted in it being targeted as a novel therapeutic agent for various pathological conditions. This broad range of actions indicates that uvaol interacts with diverse targets to achieve its therapeutic effects. However, the mechanism of how uvaol affects cells involved in the wound healing process has yet to be delineated. Here, we evaluated how uvaol affects biological responses of fibroblasts and endothelial cells involved in wound healing, as well its healing potential for cutaneous wounds, in mice.

## 2. Results

### 2.1. Effect of Uvaol on Cell Viability

**Figure 2:**
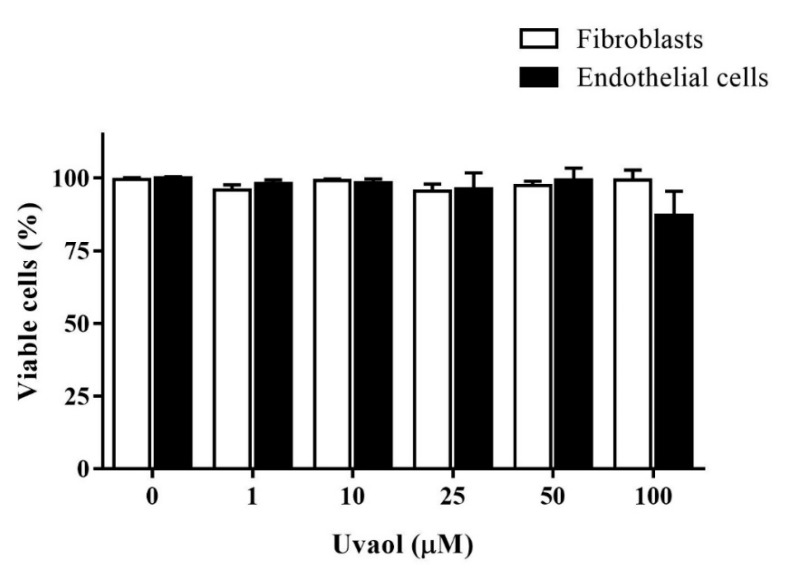
Effect of uvaol on the viability of fibroblast and endothelial cells. Cells were plated and treated with uvaol (1–100 µM) for 24 h. Cell viability was measured by MTT assay. The bars represent the mean ± standard deviation (SD) of three experiments performed in triplicate.

MTT assays were used to evaluate how uvaol affects cell viability, and to ascertain the non-toxic concentration ranges of uvaol to treat cells. There were no significant changes in the viability of fibroblasts and endothelial cells after uvaol treatment with any of the tested doses for 24 h (Figure 2). Thus, we selected the doses of 10 or 50 mM to proceed to the next step, and evaluated their respective effects on cell migration.

### 2.2. Effects of Uvaol Treatment on Fibroblast and Endothelial Cell Motility

**Figure 3:**
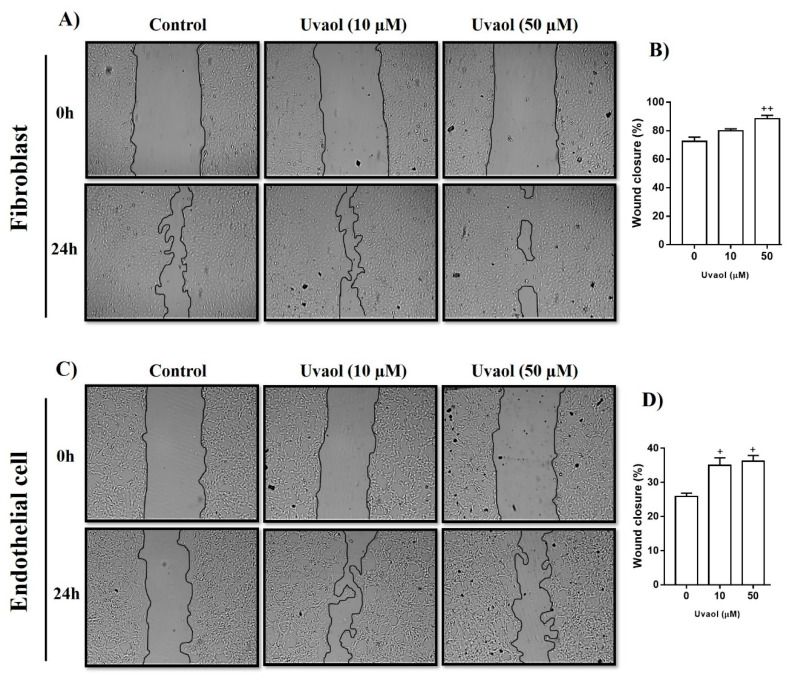
Effect of uvaol on the cell migration of fibroblasts and endothelial cells in the scratch assay. Cells were treated with 10 or 50 µM uvaol, and images were captured to calculate the scratch closure. Representative photomicrography images showing the scratched area of fibroblasts (**A**) and endothelial cells (**B**) treated with medium (control) or uvaol, and cell migration towards the cell-free area after 24 h (magnification 40×). The percentage of the scratch covered was measured by quantifying the total distance that cells moved from the edge of the scratch towards the center of the scratch, using ImageJ software, followed by conversion to a percentage of the wound that was covered (**C**,**D**). Values represent mean ± SD from three independent experiments. (+) *p* < 0.05 and (++) *p* < 0.01 compared with medium-treated cells after 24 h.

Scratch assays were used to evaluate how uvaol affected fibroblast and endothelial cell migratory activity. Scratches were made on the monolayer of confluent cells, and were then treated with the respective culture medium (control) or uvaol at concentrations of 10 or 50 μM. Cell migration to the scratch area was evaluated at 0 and 24 h. Compared to DMEM-treated cells (control), only uvaol only at 50 mM concentration accelerated the migration of fibroblasts towards the scratched area (Figure 3A), with a significant wound closure rate of 22% (Figure 3B). Compared to RPMI-treated cells (control), uvaol at both tested concentrations (10 and 50 µM) significantly increased the migration of endothelial cells towards the scratched area (Figure 3C), with significant wound closure rates of 36% and 40%, respectively (Figure 3D). Thus, uvaol directly affects fibroblast and endothelial cells by enhancing their migration.

### 2.3. Effect of Uvaol on ECM Deposition by Fibroblasts

**Figure 4:**
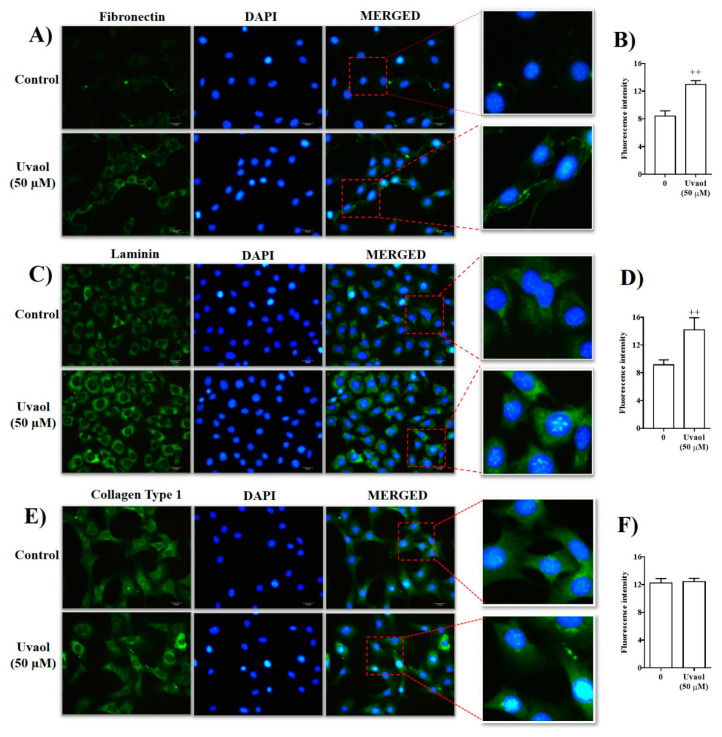
Effect of uvaol on the levels of fibronectin, laminin, and collagen type I in fibroblasts using immunofluorescence analysis. Fibroblasts were cultured with and without 50 µM uvaol. After 24 h, the cells were fixed and the extracellular matrix was immuno-stained using antibodies against fibronectin (**A**), laminin (**C**), and collagen type I (**E**). Nuclei were stained with DAPI. Each panel shows an image of one representative field from three independent experiments. Graph showing the results of the quantification of extracellular matrix synthesis of images from the respective panel (**B**,**D**,**F**). The image is displayed at × 400 original magnification and the red box indicates the region acquired for the quantification of extracellular matrix. Bars represent mean ± SD of three independent experiments. Statistical significance between groups was determined by ANOVA followed by Bonferroni’s test. (++) *p* < 0.01 compared with respective medium-treated group.

ECM contributes to various functions of cells, including migration. Thus, we evaluated whether uvaol treatment affects ECM protein synthesis. Fibroblasts were exposed for 24 h to uvaol, after which fibronectin, laminin, and collagen type I production was assessed by immunofluorescence analysis. Cells treated with DMEM (control) showed a basal level of fibronectin protein production organized around the cell nucleus (Figure 4A). Treatment with 50 µM uvaol increased the immunofluorescence staining of cytoplasmic fibronectin. Image analysis showed a 30% increase in intracytoplasmic fluorescence, reflecting fibronectin levels after treatment (Figure 4B). A similar phenomenon was observed in the production of laminin. Cells treated with DMEM (control) showed a basal level of laminin production in the cell cytoplasm (Figure 4C). Treatment with 50 µM uvaol increased the immunofluorescence staining of cytoplasmic laminin. Image analysis showed a 36% increase in laminin levels after treatment (Figure 4D). Unlike the proteins laminin and fibronectin, basal collagen type I expression in fibroblasts did not change after treatment with 50 µM uvaol for 24 h (Figure 4E,F).

### 2.4. Uvaol Stimulates Tube-Like Structure Formation In Vitro

**Figure 5:**
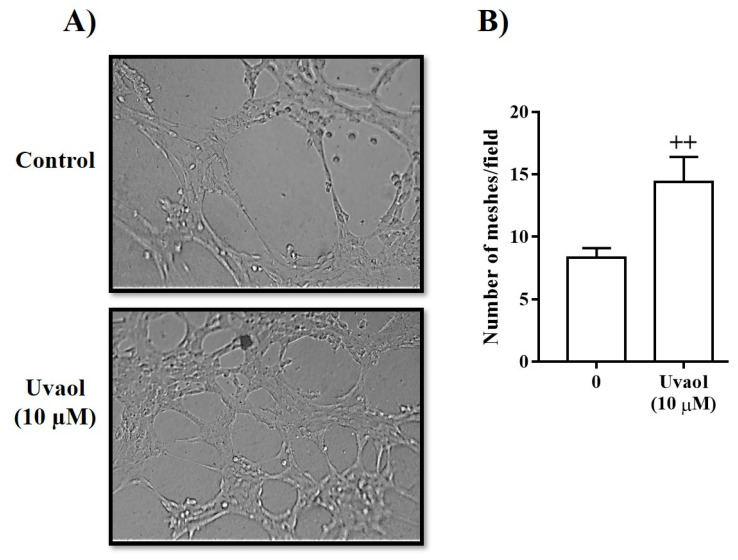
Effect of uvaol on the formation of the tubular network in endothelial cells on Matrigel after 16 h. (**A**) Representative images of tubule-like structures on Matrigel by endothelial cells following 16 h of treatment. The tubes were photographed under the microscope at 200× magnification. (**B**) Analysis of the number of meshes formed after medium or uvaol treatment. Bars represent mean ± SD of three independent experiments. Statistical significance between groups was determined by Student’s test. (++) *p* < 0.01 compared with medium-treated cells after 16 h.

To investigate whether uvaol affects endothelial morphogenesis, we employed an in vitro model of tube formation in which t.End1 cells assemble into vessel-like tubes containing lumens. Compared with the medium-treated cells (control), endothelial cells exposed to uvaol (10 µM) for 6 h exhibited an approximately 1.8-fold increase in tube-like structure formation (control) (Figure 5A,B).

### 2.5. Involvement of the PKA and p38-MAPK Signaling Pathways in Uvaol Induced both Fibroblast and Endothelial Cell Motility

**Figure 6:**
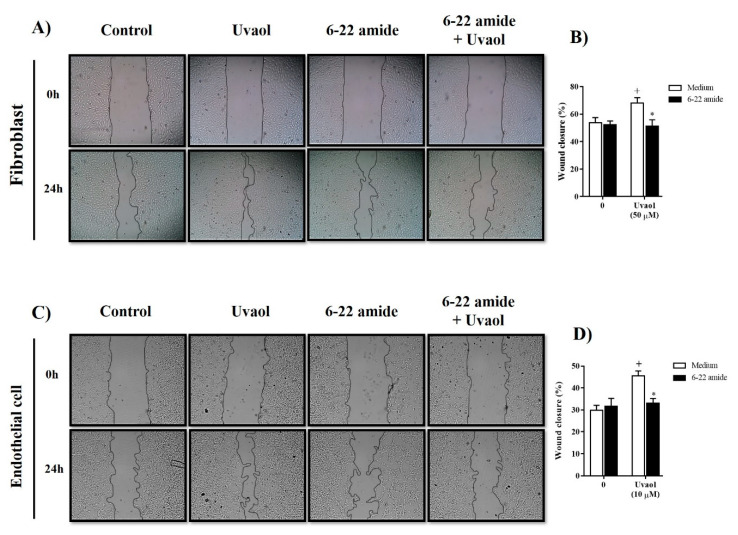
Inhibition of the PKA signaling pathway attenuates the migration of fibroblast and endothelial cells induced by uvaol. Scratch wounds were created in cell monolayers of cells using a sterile pipette tip. Representative photomicrography images showing the scratched area of fibroblasts (**A**) and endothelial cells (**C**) cultured with medium, uvaol or (6-22)-amide (a PKA inhibitor, 10 µM) in the presence of uvaol for 24 h (magnification 40×). The percentage of the scratch covered was measured by quantifying the total distance that cells moved from the edge of the scratch towards the center of the scratch, using ImageJ software, followed by conversion to a percentage of the wound that was covered (**B**,**D**). The bars represent mean ± SD of three independent experiments. Statistical differences were detected with two-way ANOVA followed by the Bonferroni test. (+) *p* < 0.01 compared to medium-treated cells, (*) *p* < 0.05 compared to uvaol-treated cells.

Because PKA and p38-MAPK cellular signaling pathways are associated with cell motility, we evaluated whether the effects of uvaol on motility of fibroblast and endothelial cells involved these protein kinases by using specific inhibitors of intracellular signaling. Compared to DMEM-treated cells (control), uvaol accelerated the migration of fibroblasts towards the scratched area after 24 h, with PKA inhibitor (PKI-(6-22)-amide (10 µM) treatment inhibiting this phenomenon by 25% (Figure 6A,B). Compared to medium-treated cells (control), uvaol accelerated the migration of endothelial cells towards the scratched area after 24 h (Figure 6C). Similar to that observed for fibroblasts, treatment with the PKA inhibitor (PKI-(6-22)-amide (10 µM) inhibited uvaol-induced endothelial cells’ migration by 27% (Figure 6D).

**Figure 7:**
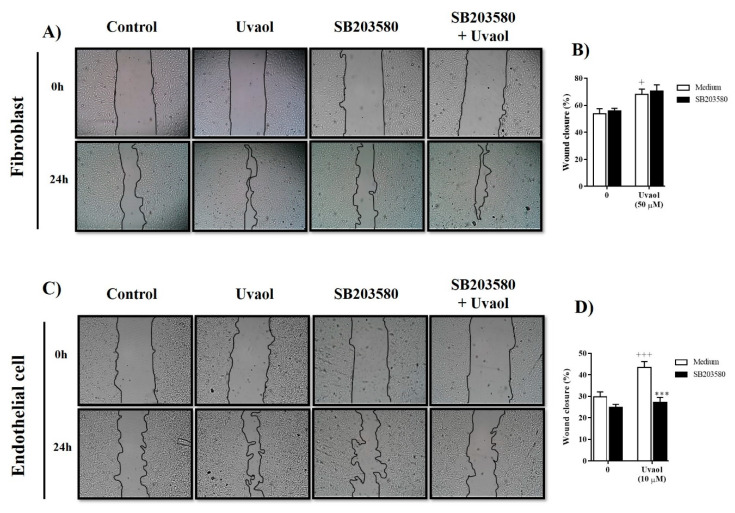
Inhibition of the p38-MAPK signaling pathway suppressed uvaol-improved migration in endothelial cells but not in fibroblasts. Scratch wounds were created in monolayers of cells using a sterile pipette tip. Representative photomicrography images showing the scratched area of fibroblasts (**A**) and endothelial cells (**C**) cultured with medium, uvaol or SB203580 (a p38-MAPK inhibitor, 10 µM) in the presence of uvaol for 24 h (magnification 40×). The percentage of the scratch covered was measured by quantifying the total distance that cells moved from the edge of the scratch towards the center of the scratch, using ImageJ software, followed by conversion to a percentage of the wound that was covered (**B**,**D**). Bars represent mean ± SD of three independent experiments. Statistical differences were detected with two-way ANOVA followed by the Bonferroni test. (+) *p* < 0.01 and (+++) *p* < 0.001 compared to medium-treated cells, (***) *p* < 0.001 compared to uvaol-treated cells.

Compared to medium-treated cells (control), uvaol accelerated the migration of fibroblasts towards the scratched area after 24 h (Figure 7A). Treatment with the p38-MAPK inhibitor (SB203580, 10 µM) did not cause any significant alteration in uvaol-induced fibroblast migration (Figure 7B). However, the accelerated migration of endothelial cells in response to uvaol treatment was significantly inhibited in 37% by the inhibitor for p38-MAPK (Figure 7C,D). Thus, the enhanced migration of endothelial caused by uvaol is associated with both PKA and p38-MAPK signaling pathways. However, uvaol-induced migration in fibroblasts seemed to be dependent on the PKA pathway but not the p38-MAPK signaling pathway.

### 2.6. Effects of Uvaol on the In Vivo Wound Healing Assay

**Figure 8:**
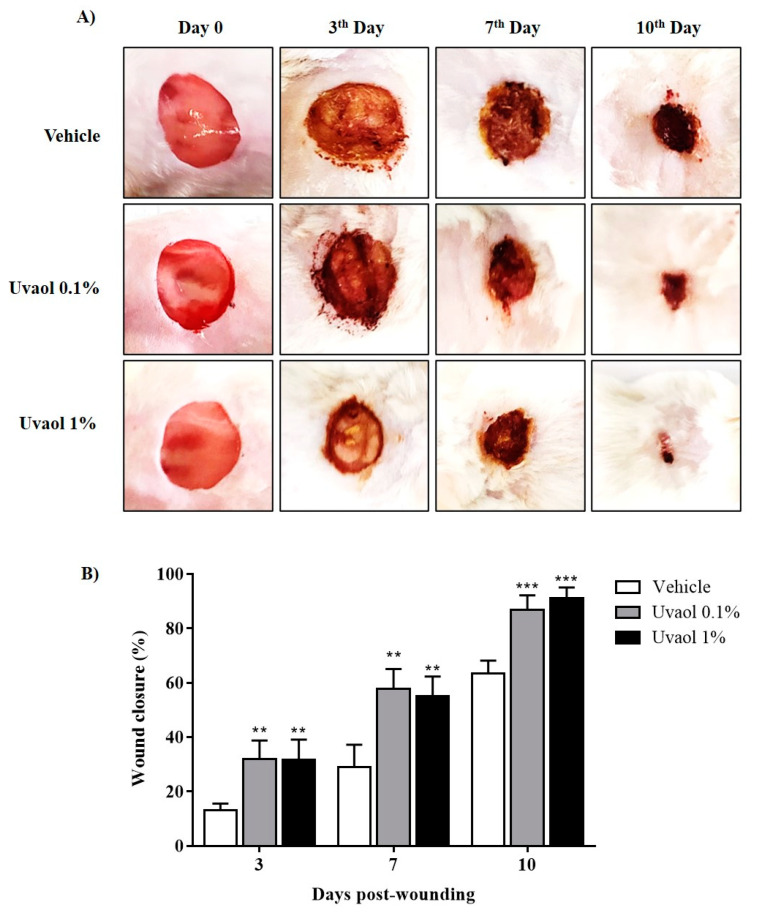
Effect of the topical application of uvaol in the excisional wounds of mice. (**A**) Representative photographs of wounds on days post wounding of animals treated with vehicle (PBS) and uvaol (0.1% or 1%). (**B**) Wound closure kinetics. The bars represent mean ± SD, *n* = 5 for each time point and group. Statistical differences were detected with two-way ANOVA followed by the Bonferroni test. (**) *p* < 0.01 and (***) *p* < 0.001 compared to vehicle-treated cells on each respective day.

During the wound healing process, deposition of the extracellular matrix and angiogenesis are necessary to form a new tissue and accelerate wound closure. Thus, we used the excision wound model to evaluate how uvaol affects wound healing in mice. The progression of healing was evaluated on days 3, 7, and 10 post wounding. The progression of wound closure after topical treatment with vehicle or uvaol (0.1% or 1%) was evaluated. In vehicle-treated animals, the wounded area reduced by 9% (day 3), 19% (day 7), and 35% (day 10) (Figure 8A). After application of 0.1% and 1% uvaol topically, wound area declined by 10% and 18% on day 3, 39% and 40% on day 7, and 59% and 60% on day 10, respectively (Figure 8B). During the healing period, the presence of infections that could extend the time of wound contraction was not detected. Thus, uvaol exhibited healing properties in vivo, in parallel to directly affecting the functioning of fibroblasts and endothelial cells in vitro.

## 3. Discussion

Triterpenes constitute a class of natural compounds that originate from plants that have a wide range of pharmacological effects. Thus, these compounds might be useful for treating various pathological conditions, including cutaneous wounds. During the healing process, certain mechanisms promote the wound healing process, including the migration of cells towards the injury area, the production of the extracellular matrix by fibroblasts, and the formation of new blood vessels by endothelial cells [2,19]. Thus, identifying compounds that improve these processes could lead to the discovery of new and cost-effective drugs for treating wounds. However, to the best of our knowledge, published studies are not available on how uvaol affects the functions of fibroblast and endothelial cells in vitro. Additionally, information remains limited on how uvaol influences cutaneous wound healing. This study demonstrated in vitro that uvaol accelerates cell migration via signaling pathways dependent on both PKA and p38-MAPK. We also demonstrated that it stimulated the production of ECM from fibroblasts, and the formation of tube-like structures from endothelial cells. Moreover, we confirmed that uvaol topical treatment accelerated the healing of cutaneous wounds.

Wound healing is characterized by the production of the extracellular matrix, which is associated with angiogenesis, requiring both fibroblasts and endothelial cells to complete the proliferative and remodeling phases [20]. During the course of the wound healing process, fibroblasts secrete extracellular matrix proteins that help the underlying endothelial cells to proliferate rapidly and migrate to the wound bed to form new vessels and ensure sufficient nutrients and oxygen reach the wound area to form new tissue [21]. Of note, healing is hindered if these cells fail to function, as observed in various non-healing diseases, such as diabetes and chronic venous leg ulcers [22]. Yet, these observations reinforce that stimuli with the ability to improve fibroblast and endothelial cell functions have a positive effect on wound healing [23].

One possible effect of uvaol on the viability of fibroblast and endothelial cells was evaluated to avoid the use of cytotoxic concentrations. We showed that uvaol does not have cytotoxic effects on fibroblasts or endothelial cells after 24 h of exposure at dose ranges of 1 to 100 µM. This result confirms previous studies that demonstrated the absence of toxicity of uvaol to distinct cell types [24]. Of note, uvaol prevents cell death caused by bacterial products [25], and has protector effects against heavy metal-induced cytotoxicity [26].

We subsequently assessed the influence of uvaol on cell migration using a scratch assay. Scratch assays are a standard in vitro methodology routinely used to identify substances that regulate cellular migration [27]. Our results showed that uvaol accelerated the migration of fibroblasts and endothelial cells. Thus, this triterpene directly affects these cells. These results were consistent with previous studies in which another triterpene also enhanced the motility of fibroblast and endothelial cells [28,29]. It is extremely important for these cells to migrate during the granulation tissue formation phase of wound healing [30].

We subsequently evaluated whether uvaol affects cellular events associated with wound healing, such as the deposition of ECM proteins and angiogenesis. Our study showed that the synthesis of fibronectin and laminin is enhanced in uvaol-treated fibroblasts, without impacting the production of type I collagen. Our results supported previous studies, which showed that other triterpenes and natural products, such as ginsenoside and propolis, enhance the synthesis of extracellular matrix proteins [31,32,33]. Furthermore, a pro-angiogenic effect of uvaol was observed in endothelial cells. In the tube formation assay, uvaol-treated cells clearly enhanced tube formation. This result indicates the potential of uvaol at inducing the formation of new blood vessels from existing vascular structures during angiogenesis. Our findings support previous studies that reported the pro-angiogenic effect of other triterpenes, such as asiaticoside [34], betulinic acid [35], astragaloside IV [36], and lupeol [29]. Overall, our results indicate that uvaol enhances the functions of fibroblasts and endothelial cells, which could be useful for wound healing.

The migration of fibroblast and endothelial cells is mediated by multiple intracellular signaling pathways, including protein kinase A (PKA) and p38-MAPK pathways [37]. Even knowing that uvaol downregulates the AKT/PI3K signaling pathway in cancer cells, such as HepG2 cells [38], knowledge remains limited about the effect of uvaol on signaling pathways of fibroblast and endothelial cells. Thus, to evaluate whether uvaol has an activating effect on these intracellular signaling pathways, we evaluated the migration of fibroblast and endothelial cells using the scratch assay in the presence of inhibitors of distinct signaling pathways. Our study demonstrated that the inhibitor PKI-(6-22)-amide prevented the migration of uvaol-provoked fibroblast and endothelial cells; thus, this PKA signaling pathway is likely involved in uvaol-induced migration. These findings support previous studies, which showed that other triterpenoids, such as ursolic acid [39], astragaloside [40], and fernenediol [41], induce the activation of the PKA pathway to generate their pharmacological effects. Of note, the activation of PKA intracellular signaling pathways upregulates ECM production by fibroblasts, contributing to tube formation and the motility of endothelial cells [42,43]. Our results revealed that the uvaol-induced migration of endothelial cells, but not fibroblasts, was inhibited by the inhibitor SB203580 in the p38-MAPK signaling pathway. Previous studies have already demonstrated that p38-MAPK pathway activity in endothelial cells is important for organizing cells into tube-like structures [44]. Thus, uvaol stimulates the activation of PKA and p38-MAPK signaling pathways in endothelial cells but only in the PKA signaling pathway for fibroblasts.

Wound healing is a dynamic process, composed of a cascade of interlocking biological events, in which the transition from inflammation to subsequent phases may be one of the most critical and defining steps. There is evidence to suggest that persistent inflammation results in upregulation of various proinflammatory cytokines and chemokines, which leads to delay in the wound closure [45]. Additionally, it has been reported that an excessive and prolonged inflammatory phase may be a factor in the conversion of acute wounds into non-healing chronic wounds [46]. Thus, active compounds able to reduce the intensity of the inflammatory response might accelerate the healing process by shortening the inflammatory phase. Previous studies reported that uvaol had shown anti-inflammatory activity by suppressing the secretion of inflammatory cytokines [16,47]. Inhibition of proinflammatory mediators may regulate the progress of cutaneous wound healing and thus represent a good therapeutic target [48]. Therefore, although speculative, the idea of uvaol accelerating wound closure by shortening the inflammatory phase cannot be dismissed. Further studies are necessary to investigate this concept.

This study showed that uvaol exhibits pharmacological effects that improve the functions of both fibroblasts and endothelial cells; thus, we evaluated whether uvaol accelerates the closing of cutaneous wounds. Our macroscopic analysis revealed that the topical application of uvaol promotes the healing of skin wounds by accelerating the closure of lesions from day 7 post-wounding. Based on our results and those of previous studies, uvaol might act by accelerating each phase of the healing process in the wound microenvironment of cells. This phenomenon might result in the faster closure of wounds.

In conclusion, the current study demonstrated that uvaol stimulates cellular events in fibroblast and endothelial cells that are critical for wound healing. This stimulatory activity supports the application of uvaol as a therapeutic agent in cutaneous wound healing.

## 4. Materials and Methods

### 4.1. Reagents

The following substances, purchased from Sigma Chemical Co. (St. Louis, MO, USA), were used: uvaol (Urs-12-ene-3,28-diol, ≥95% purity, PubChem CID: 92802; Figure 1), Tween-20, eosin, hematoxylin, Dulbecco’s Modified Eagle Medium (DMEM), Roswell Park Memorial Institute medium (RPMI-1640), 3-[4,5-dimethylthiazol-2-yl]-2,5-diphenyltetrazolium bromide (MTT), phosphate-buffered saline (PBS) gentamicin, fetal bovine serum (FBS), trypsin, l-glutamine, 4′,6-diamidino-2′-phenylindole dihydrochloride (DAPI), SB203580 (p38-MAPK inhibitor), and (6-22)-amide (inhibitor of PKA). Xylazine (Anasedan^®^) and ketamine (Dopalen^®^) from Ceva (Paulínia, São Paulo, BRA), ethylenediaminetetraacetic acid (EDTA) and dimethyl sulfoxide (DMSO) from Synth (Diadema, SP, BRA). Antibodies against laminin, fibronectin, and collagen type I were purchased from Novotec (Bron, Lyon, France), while anti-goat IgG-fluorescein isothiocyanate (FITC) was from Santa Cruz Biotechnology (Dallas, TX, USA). Stock solution of uvaol was prepared in DMSO. The DMSO concentration applied to cells in culture never exceeded 0.1%. Neither the vehicle nor any of the compounds used in this study altered cell viability.

### 4.2. Cell Culture

The NIH3T3 fibroblast cell line and endothelioma tEnd.1 cell line were provided by Cell Bank of Rio de Janeiro, being maintained in DMEM and RPMI-1640, respectively. Both types of media were supplemented with 10% of heat-inactivated fetal bovine serum (FBS), l-glutamine (2 mM), and gentamicin (40 µg/mL) at 37 °C and in a humidified atmosphere containing 5% CO_2_. The assays were performed using cells between three and six passages. In all experiments, untreated cells were used as negative controls.

### 4.3. Cell Viability Assay

The MTT assay was used to evaluate how uvaol affected cell viability [49]. In brief, NIH3T3 fibroblasts (7 × 10³ cells/well) and tEnd.1 cells (10^4^ cells/well) were seeded in 96-well plates and treated with uvaol at concentrations of 1, 10, 25, 50, and 100 µM for 24 h. Thereafter, the medium was replaced with fresh medium containing 5 mg/mL MTT (3-(4,5-dimethylthiazol-2-yl)-2,5-diphe-nyltetrazolium bromide). Following incubation (4 h) in a humidified CO_2_ incubator at 37 °C and 5% CO_2_, the supernatant was discarded and dimethyl sulfoxide solution (DMSO, 150 μL/well) was added to each well to solubilize the formazan crystals that had formed. After incubation at room temperature for 15 min, the optical density (OD) was measured at 540 nm using a spectrophotometer. Three separate wells were assayed for each treatment. The percentage viability relative to the control sample was determined as (OD of treated cells/OD of untreated cells) × 100.

### 4.4. Scratch Wound Healing Assay

To evaluate how uvaol affects fibroblast motility, we used the scratch assay, as described by [50]. Cells were cultured in a 24-well plate using medium containing 1% FBS, until 90% confluency was reached. Thereafter, a vertical stripe on the cell monolayer was made using a sterile pipette (200 μL) tip. The wells were washed twice with PBS to remove cell debris. Then, uvaol was added at concentrations of 1, 10, and 50 μM. As a control, the cells were treated with DMEM or RPMI containing 1% FBS. A low concentration of serum is the most common non-pharmaceutical method for minimizing proliferation in wound healing assays [51].

In a second set of experiments, the scratched monolayer was treated with the inhibitor of cAMP-dependent protein kinase A (PKA) (fragment (6-22)-amide, 10 µM) or with the inhibitor of p38 mitogen-activated protein kinase (p38-MAPK) (SB203580, 10 µM) 1 h before incubation with uvaol. After 24 h of treatment, the cells were photographed using invert microscopy (Olympus IX70, Tokyo, Japan) with a digital camera to measure wound closure (magnification, 40×). Cell migration was analyzed using ImageJ software (version 1.51k), and was expressed as the percentage of closure of the initial wound (scratch width at time zero) using the following formula: [(scratch width at time 0 − scratch width after an identified culture period)/(scratch width at time 0)] × 100%.

### 4.5. Immunofluorescence Staining

Fibroblasts (7 × 10^3^) were cultured in 8-well Lab-Tek™ chamber glass slides (Thermo scientific) (Waltham, Massachusetts, USA) with complete medium (DMEM) for 24 h. The medium was then replaced, and the cells were treated with 50 µM uvaol for 24 h. Cells maintained in DMEM under the same conditions were used as controls. After treatment, the cultures were washed with PBS, fixed with 100% methanol for 10 min, and subjected to an indirect immunofluorescence assay, as previously described [52]. In brief, cells were rehydrated in PBS and incubated for 1 h with PBS containing 1% BSA to block non-specific binding. Next, samples were incubated with primary specific anti-fibronectin, anti-laminin, or anti-collagen type I antibodies (1:50) for 1 h in a humidified chamber. They were then washed with PBS, and incubated with appropriate FITC-conjugated secondary antibody (1:200) for 45 min at room temperature. DAPI staining was used to visualize the nuclei. Immuno-stained samples were analyzed by fluorescence microscopy. The staining of each section was analyzed to obtain the mean optical density value (MOD), which represented the fluorescence intensity per pixel [53].

### 4.6. Tube-Like Structure Formation Assay

The Matrigel-based tube formation assay was used to evaluate the angiogenic potential of uvaol. In brief, 80μL Matrigel solution (BD Biosciences) was applied to 98-well plates 2 h before cell seeding. After matrix formation, endothelial cells (104 cells) were placed on the matrix gel and cultured in the presence or absence of uvaol (10 µM) with RPMI medium containing 2% serum at 37 °C in a 5% CO_2_ incubator. After 16 h, the formed network of tubes was visualized at 100× magnification by light microscopy. The number of tube-like structures per field was measured using angiogenesis Analyzer plugin in ImageJ. Each experiment was performed in triplicate. All data analyses were performed in a blinded manner.

### 4.7. Animals

Experiments were carried out on adult male Swiss mice (25–35 g) obtained from the Federal University of Alagoas (UFAL), Brazil, breeding unit. The animals were maintained with free access to food and water. They were kept at 22–28 °C, with a controlled 12 h light/dark cycle at the Institute of Biological and Health Sciences (UFAL). Experiments were performed during the light phase of the cycle. All experiments were carried out in accordance with institutional guidelines and ethics (License Number 016/2014).

### 4.8. Mouse Excisional Wound Model

The animals were anesthetized with an intraperitoneal (i.p.) injection of ketamine and xylazine (25 mg/kg, 25 mg/kg). They were shaved at the predetermined site before wounding. Subsequently, the dorsal region was shaved, wiped topically with distilled water, and circular wounds were made using a template of metal circle with a diameter of 1 cm. Animals were housed separately in disinfected cages after recovery from anesthesia. These conditions were maintained throughout the experiments [54]. The animals were then placed in separate cages to avoid any disturbance. Animals were topically treated with 50 μL of saline solution (vehicle, NaCl, 0.9%) or 50 μL of uvaol at 0.1% for 1% for once a day for 10 consecutive days starting from the day of wounding. All animals received topical treatment on the wounds once daily until the end of the experiments.

### 4.9. Wound Contraction Measurements

Macroscopic evaluation of the wounds was performed using a digital camera on day 0 (before the start of treatment) and on days 3, 7, and 10 after injury. The Optical SteadyShot DSC-W350 digital camera (14.1 megapixels) was positioned at a distance of 15 cm of wound. The data were analyzed using ImageJ software. The wound measurements on various days were expressed as the percentage of wound closure. The values were expressed as percentage values of day 0 measurements, and were calculated by the formula: [(A0 − AI)/A0 × 100], where A0 is the initial wound area (day 0) and AI is the wound area on days 3, 7, and 10 after the initial wound.

### 4.10. Statistical Analysis

Data were reported as mean ± standard deviation (SD), and were analyzed using GraphPad Prism software, version 5.0 (San Diego, CA, USA). One-way analysis of variance (ANOVA) followed by the post hoc Bonferroni test multiple comparison test was applied for the comparison among various groups and Student’s t test to determine the significance of differences between two means. *p* values less than 0.05 were considered statistically significant.
